# Plasticity of Carbohydrate Transport at the Blood-Brain Barrier

**DOI:** 10.3389/fnbeh.2020.612430

**Published:** 2021-01-22

**Authors:** Ellen McMullen, Astrid Weiler, Holger M. Becker, Stefanie Schirmeier

**Affiliations:** ^1^Department of Biology, Institute of Zoology, Technische Universität Dresden, Dresden, Germany; ^2^Division of General Zoology, Department of Biology, University of Kaiserslautern, Kaiserslautern, Germany

**Keywords:** blood-brain barrier, carbohydrate transport, compensatory mechanisms, transporter regulation, transport dynamics

## Abstract

Neuronal function is highly energy demanding, requiring efficient transport of nutrients into the central nervous system (CNS). Simultaneously the brain must be protected from the influx of unwanted solutes. Most of the energy is supplied from dietary sugars, delivered from circulation via the blood-brain barrier (BBB). Therefore, selective transporters are required to shuttle metabolites into the nervous system where they can be utilized. The Drosophila BBB is formed by perineural and subperineurial glial cells, which effectively separate the brain from the surrounding hemolymph, maintaining a constant microenvironment. We identified two previously unknown BBB transporters, MFS3 (Major Facilitator Superfamily Transporter 3), located in the perineurial glial cells, and Pippin, found in both the perineurial and subperineurial glial cells. Both transporters facilitate uptake of circulating trehalose and glucose into the BBB-forming glial cells. RNA interference-mediated knockdown of these transporters leads to pupal lethality. However, null mutants reach adulthood, although they do show reduced lifespan and activity. Here, we report that both carbohydrate transport efficiency and resulting lethality found upon loss of MFS3 or Pippin are rescued via compensatory upregulation of Tret1-1, another BBB carbohydrate transporter, in *Mfs3* and *pippin* null mutants, while RNAi-mediated knockdown is not compensated for. This means that the compensatory mechanisms in place upon mRNA degradation following RNA interference can be vastly different from those resulting from a null mutation.

## Introduction

To allow full functionality the brain requires a lot of energy. Most of the energy used in the nervous system is gained via carbohydrate metabolism. The human adult brain, despite accounting for only 2% of the bodies overall mass, consumes ~20% of the total oxygen (Mink et al., 1981; Laughlin et al., 1998; Harris et al., 2012). The oxygen is used to metabolize large amounts of glucose. The human brain uses about 90 g of glucose per day; during childhood carbohydrate usage is even higher (Kuzawa et al., 2014). Likewise, the blowfly retina consumes ~10% of the total ATP produced, which is close to the consumption observed in vertebrates (Laughlin et al., 1998).

Neuronal activity also relies on a tightly regulated extracellular milieu to allow signal conductance. Thus, the brain is shielded from potentially harmful substances, like high, and fluctuating ion concentrations found in circulation, by the blood-brain barrier (BBB). In mammals, the endothelial cells forming brain capillaries build intercellular tight junctions that prevent paracellular diffusion, thereby uncoupling the brain from circulation. In addition, efflux transporters of the ABC family transport lipophilic, membrane-permeable molecules out of the BBB-forming cells to protect the nervous system form neurotoxic substances (for reviews see Löscher and Potschka, 2005; Koehn, 2020). To allow sufficient nutrient supply, a variety of transport proteins are expressed in the endothelial cells (for a review on metabolite transport at the BBB, see Weiler et al., 2017). In mammals, Glut1 is the main carbohydrate transporter found in the BBB-forming cells. Two differently glycosylated isoforms of Glut1 have been found in the mammalian nervous system, a 45 kDa and a 55 kDa isoform, that show identical transport kinetics (Birnbaum et al., 1986; Sivitz et al., 1989). The 55 kDa isoform is exclusively expressed in the endothelial cells and localizes to the luminal and abluminal membranes, while the 45 kDa isoform is found in astrocytes (Dick et al., 1984; Gerhart et al., 1989; Sivitz et al., 1989; Harik et al., 1990; Farrell and Pardridge, 1991; Maher et al., 1991, 1994; Simpson et al., 2001). In addition to Glut1, sodium glucose cotransporters (SGLTs) are expressed in the BBB upon stress. SGLT1 and SGLT2 have been shown to be expressed upon oxygen deprivation or ischemia, but seem to play a minor role in glucose uptake under normal conditions (Nishizaki et al., 1995; Nishizaki and Matsuoka, 1998; Elfeber et al., 2004; Enerson and Drewes, 2006; Vemula et al., 2009; Yu et al., 2013). Interestingly, the abundance of GLUT1 in the BBB seems to be regulated by hypoglycemia (Boado and Pardridge, 1993; Kumagai et al., 1995; Simpson et al., 1999). However, the regulatory mechanisms that underlie transporter regulation in the mammalian BBB are unknown.

In Drosophila, as in mammals, the brain is shielded from circulation. Here, the BBB is formed by two layers of glial cells, the subperineurial glial cells and the perineurial glial cells that surround the entire nervous system (reviewed in Limmer et al., 2014; Yildirim et al., 2019). Insects possess an open circulatory system, thus all organs, including the brain, are floating in the hemolymph. Therefore, the BBB surrounds the entire nervous system like a sheath. The subperineurial glial cells form intercellular pleated septate junctions that prevent paracellular diffusion (Stork et al., 2008). As in mammals, efflux transporters protect the nervous system from lipid-soluble toxic substances (reviewed in Hindle and Bainton, 2014). To ensure sufficient supply of nutrients and other essential substances to the nervous system a variety of solute carrier family transporter proteins are expressed in the BBB (Desalvo et al., 2014; Weiler et al., 2017). In addition, carbohydrate transporters are required to provide a sufficient supply of carbohydrates to the nervous system. As well as glucose, the non-reducing disaccharide trehalose is found in high quantities in circulation in Drosophila (Wyatt and Kalf, 1957; Lee and Park, 2004; Broughton et al., 2008; Pasco and Léopold, 2012). It has been shown that glucose can be readily taken up into the nervous system (Volkenhoff et al., 2018). Furthermore, the trehalose transporter 1-1 (Tret1-1) is expressed specifically in the perineurial glial cells of the Drosophila BBB (Volkenhoff et al., 2015). Tret1-1 is homologous to mammalian GLUT6 and GLUT8 and has been shown to transport trehalose and glucose (Kanamori et al., 2010; Hertenstein et al., 2020). How carbohydrates are taken up into the subperineurial glial cells of the BBB and the other neural cells in the Drosophila nervous system is currently unknown. There are several homologs of mammalian GLUT1 encoded in the Drosophila genome: the closest homologs are dmGlut1, dmSut1 (sugar transporter 1), dmSut2, dmSut3, and CG7882. dmGlut1 is specifically expressed in neurons and may facilitate carbohydrate uptake there (Volkenhoff et al., 2018). Transcriptomic and *in situ* data for CG7882 and dmSut1-3, indicate very little or no expression in the nervous system, suggesting no major role in neural carbohydrate transport (Weiszmann et al., 2009; Croset et al., 2018; Davie et al., 2018).

Here, we identify two additional carbohydrate transporters expressed in the Drosophila BBB, Major Facilitator Superfamily Transporter 3 (MFS3, CG4726) and Pippin (CG4797). Pippin is expressed in both perineurial and subperineurial glial cells, while MFS3 is expressed in the perineurial glial cells only. Both transporters are able to facilitate uptake of glucose and trehalose when heterologously expressed in *Xenopus laevis* oocytes. Likewise, the simultaneous loss of Pippin and MFS3 in perineural glia, and Pippin alone in subperineurial glia leads to decreased uptake of glucose. Interestingly, loss of either transporter or both transporters does not have any major phenotypic consequences. We demonstrate here that in null mutants compensatory upregulation of Tret1-1 rescues the detrimental effects of acute transporter loss on viability and carbohydrate transport at the BBB, while RNAi-mediated knockdown is not compensated for. In summary, we show that expression of carbohydrate transporters in the Drosophila BBB is highly dynamic and can be adapted to suboptimal circumstances like loss of one transporter. This dynamic adaptation of carbohydrate transport can most likely also be used to spare the nervous system from effects of hypoglycemia or malnutrition.

## Materials and Methods

### Fly Stocks

Flies were kept at room temperature or 25°C. The following fly lines were used w^−^;nrv2-Gal4;nrv2-Gal4, apontic-Gal4, mCherry^dsRNA^ (BL35785), UAS-CD8-GFP, nanos-Cas9^attP2A^ (BL36046) (Bloomington Drosophila stock center), *PBac{681.P.FSVS-1}MFS3*^*CPTI002305*^ (Kyoto stock center), pippin-dsRNA: w^1118^; P{GD4548}^v10598^ (VDRC), repo-Gal4; repo-Gal4, alrm-Gal4; alrm-Gal4, gli-Gal4 (Christian Klämbt), moody-Gal4 (Stork et al., 2008), 46F-Gal4 (Hummel et al., 2002), MFS ^*dsRNA4726R*−3^ (Japanese National Institute of Genetics), UAS-FLII^12^Pglu-700μδ6 (Volkenhoff et al., 2018). The dsRNA-constructs used in the RNAi screen are indicated in Supplementary Table 1 and were obtained from Bloomington Drosophila stock center, VDRC or the National Institute of Genetics (NIG).

### RNA Interference Screen

The RNAi screen was performed as follows: dsRNA lines were crossed to repo-Gal4; repo-Gal4 for panglial dsRNA expression. Crosses were kept at 25°C throughout development. After 2 weeks, viability of the offspring was determined and, if available, 20 female flies were selected, and locomotor capacity was tested in the island assay 1 week later (Schmidt et al., 2012).

### RNA Interference Viability Assessment

*pippin*^*dsRNA10598*^ and *MFS3* ^*dsRNA4726R-3*^ were first crossed with a panglial driver (repo-Gal4; repo-Gal4). Crosses were performed at 25°C, after 2 weeks the viability of the offspring was determined. This method was repeated using glial subtype specific Gal4 drivers.

### Analysis of Sugar Transport Capacity in *Xenopus laevis* Oocytes

Oocytes were isolated from female *Xenopus leavis* frogs as previously described (Becker et al., 2004; Becker, 2014). The procedure was approved by the Landesuntersuchungsamt Rheinland-Pfalz, Koblenz (23 177-07/A07-2-003 §6). *D. melanogaster pippin* and *Mfs3* were first cloned into a pUASTattBrfa3xHA vector (Rodrigues et al., 2012). Afterwards, the coding sequence with the C-terminal 3xHA-tag was cloned into a pGEM-He-Juel vector. cRNA was synthesized by *in vitro* transcription using the mMESSAGE mMACHINE® T7 Kit (Fisher Scientific). Oocytes of the developmental stages V and VI were injected with 18–20 ng of cRNA. Measurements were conducted 3–6 days after injection. Expression of Pippin-3xHA and MFS3-3xHA on the surface of oocytes was confirmed by immunohistochemistry using an anti HA antibody (Covance).

82.5 mM NaCl, 2.5 mM KCl, 1 mM CaCl_2_, 1 mM MgCl_2_, 1 mM Na_2_HPO_4_, 5 mM HEPES. Transport capacity for trehalose, glucose and fructose was determined using ^14^C-labeled sugar in oocyte saline (82.5 mM NaCl, 2.5 mM KCl, 1 mM CaCl_2_, 1 mM MgCl_2_, 1 mM Na_2_HPO_4_, 5 mM HEPES, pH 7.2) at a concentration of 0.15 μCi/100 μl. ^14^C_12_-trehalose was purchased from Hartmann Analytic, Braunschweig (#1249), ^14^C_6_-glucose and ^14^C_6_-fructose were purchased from Biotrend, Köln (#MC144-50 and 66 #MC1459-50). For each experiment 95 μl of sugar substrate was added to a batch of 6-8 oocytes and incubated for 60 min. Cells were washed four times with 4 ml of ice-cold oocyte saline. Individual cells were transferred to Pico Prais scintillation vials (Prekin Elmer) and lysed in 200 μl 5% SDS by shaking at 190 rpm for 30 min at 20–28°C. Three milliliters of Rotiszint® eco plus scintillation cocktail (Carl Roth) was added to each vial and scintillation was measured using a Tri-Carb 2810TR scintillation counter (Perkin Elmer).

Transport-mediated substrate uptake was determined by subtracting the uptake in native oocytes from the uptake in Pippin or Mfs3-expressing cells. Significance in difference was calculated using a one-tailed *T*-test or the Mann–Whitney-*U*-test for analysis of non-uniformly distributed samples.

### Generation of CRISPR Mutants

Null mutants were generated using CRISPR-mediated homologous recombination. The sgRNA target sequences (*Mfs3*: sgRNA1: GGATATATAGGCCTTACTG, sgRNA2: AATGAATTCGCTATTCAGGG; *pippin*: sgRNA1: GGTAGCATATAGTAGGGGC, sgRNA2: CGAGTCTAGGGCGACTACG) were cloned into a pCFD3-dU6:3gRNA vector (Addgene). To generate the homology construct, the mini-white coding sequence flanked by homology arms (about 1.5 kb upstream and downstream of the coding sequence of either *Mfs3* or *pippin*) was cloned into a pCR-Blunt (Thermofisher) backbone using Golden Gate cloning (Engler et al., 2008) (primers to amplify homology regions from genomic DNA: Mfs3: upstream homology arm: CCACTGCAAATGGGGAAG and CTGCCGAATGCTAAT, downstream homology arm: CCCTGAATAGCGAATTCATTG and GGTCCAAGTGCAGCGTCT; pippin upstream homology arm: TCAATGGCAAAATGACG and CCTATTATCAAGGTGC, downstream homology arm: CGTAGTCGCCCTAGACTC and CCCAAAGCTCAACCAAC). The sgRNA vectors together with the homology construct were injected into nanos-Cas9^attP2A^ embryos to induce homologous recombination.

### Generation of Pippin-HA Minigene

The gene locus (including 2.2 kb upstream and 0.5 kb downstream of the coding sequence) of pippin was assembled and C-terminally 3xHA-tagged using Golden Gate cloning (Engler et al., 2008). The assembled locus was inserted into a pUAST attB rfa vector (Stephan et al., 2008) using XbaI and HindIII restriction sites (thereby removing the UAS cassette). The resulting vector was integrated into the fly genome at landing site 86Fb.

### Age Matching of Flies for Lifespan and Activity Monitoring

Flies of the deserved genotype were placed in cages with an apple juice agar plate. After 24 h, plates were exchanged and left overnight. Embryos were washed from the plate with PBS and collected using a Pasteur pipette. Embryos were transferred into vials containing standard food. Vials were kept at 18°C for 3 weeks and adult females were collected.

### Survival Analysis

Female flies were kept in batches of 20 at 25°C throughout the experiment. Flies were flipped three times a week onto fresh food, deaths were counted. Survival rates were determined using the Kaplan-Meier approach. *P*-values were calculated using Log Rank test.

### Analysis of Locomotive Activity (DAM)

Female flies were sorted into vials of 20 and aged at 25°C for 2 or 5 weeks. Single flies were sorted into tubes containing standard food and loaded into a Drosophila activity monitor (DAM). Monitors were placed in an incubator with a 12-h light dark cycle and activity was recorded. The activity over 24 h was determined by the number of beam crosses made by the animal in this time period. *P*-values for significance were determined using Mann–Whitney rank sum test.

### Analysis of Escape Response (RING Assay)

Female flies were kept in batches of 20 and aged at 25°C for 2 or 5 weeks. Flies were transferred into negative geotaxis tubes and loaded into the RING apparatus in groups of 10 (Gargano et al., 2005). Tubes were dropped from a height of 30 cm to initiate climbing response. This was repeated five times with a 30 s break between drops to allow flies to recover. The position of the flies in the tubes was captured in digital images and the mean velocity of the flies was determined. *P*-values for significance were determined using Mann–Whitney rank sum test.

### Analysis of Circulating Glucose Levels (Glucose GO kit)

Fifteen adult female flies were collected, and a puncture was made in the thorax of each fly using forceps. Flies were then transferred to a 0.5 ml tube (containing a small hole in the base) that was placed in a 1.5 ml Eppendorf tube. Tubes were centrifuged at 13,000 RPM for 5 min at 4°C. The supernatant was collected and transferred to a new Eppendorf tube. Hemolymph was heat-inactivated at 80°C for 10 min to abolish endogenous enzymatic activity, cooled and 25 μl of buffer A (5mM Tris- HCL (pH 6.6), 137 mM NaCl, 2.7 mM KCL) was added. Glucose levels were determined using a Glucose (GO) assay kit (Sigma-Aldrich) according to the manufacturer's instructions. Difference between the control and null mutants was assessed using a one-tailed *t*-test.

### Immunohistochemistry

Wandering third instar (L3) larval or adult brains were dissected and stained following standard protocols. Samples were imaged using a Zeiss LSM 880 (Zeiss, Oberkochen, Germany). The following antibodies were used: guinea pig anti-Tret1-1 PA (1:50, Volkenhoff et al., 2015), mouse anti-NC120 (1:2 Hybridoma), rabbit anti-laminin gamma (1:1,000 Abcam A47651), Chicken anti-GFP (1:500, Aves Labs), mouse anti-HA (1:1,000 Covance). Tret1-1 fluorescence was determined by comparing the mean gray values of Tret1-1 staining of null mutants or knockdown animals to the respective control. N is the number of independent experiments; n is the total number of animals analyzed.

### Measurement of Glucose Uptake (FRET)

Null mutants or dsRNA lines were crossed with flies expressing *UAS-FLII*^*12*^*Pglu-700*μδ*6* FRET glucose sensor under the control of either apt-Gal4 or moody-Gal4. Larval brains of the desired genotype were dissected in HL3 buffer (70 mM NaCl, 5 mM KCl, 20 mM MgCl_2_, 10 mM NaHCO_3_, 115 mM sucrose, 5 mM trehalose, 5 mM HEPES; pH 7.2; ca. 350 mOsm) and attached to Poly-D-Lysine-coated coverslips. Samples were then mounted in a custom-made flow through chamber and secured to a Zeiss LSM 880 (Zeiss, Oberkochen, Germany). Buffer exchange was facilitated using a mini-peristaltic pump (MPII, Harvard Apparatus). Fluorescent images were captured using a 20x/1,0 DIC M27 75mm emersion objective (Zeiss, Oberkochen, Germany) directly after dissection. An excitation of 436/25 nm, beam splitter 455 nm, emission 480/40 nm (CFP channel); excitation 436/25 nm, beam splitter 455 nm, emission 535/30 nm (YFP channel) was used. Each brain was imaged in an independent experiment (*n* = 8–12). After 2.5 min HL3 buffer was replaced with 10 mM glucose buffer (HL3 supplemented with glucose; pH 7.2) then exchanged back to HL3 after 9 min. Data analysis was performed by generating a ROI containing the larval brain and calculating the mean gray value, minus background. N is the number of independent experiments; n is the total number of animals analyzed. Statistical regression and analysis was carried out using SigmaPlot software (Jandel). The rate of glucose uptake was calculated by selecting 10 consecutive timepoints at the beginning of the slope. The volume of glucose entering the cell was determined by the mean difference between the baseline and the maximum plateau (10 mM glucose). Statistical differences were calculated using a Mann–Whitney Rank Sum test (pairs). *P* < 0.05 were considered as significant.

## Results

### CG4797 (Pippin) and Mfs3 Encode Putative Carbohydrate Transporters of the BBB

**Figure 1:**
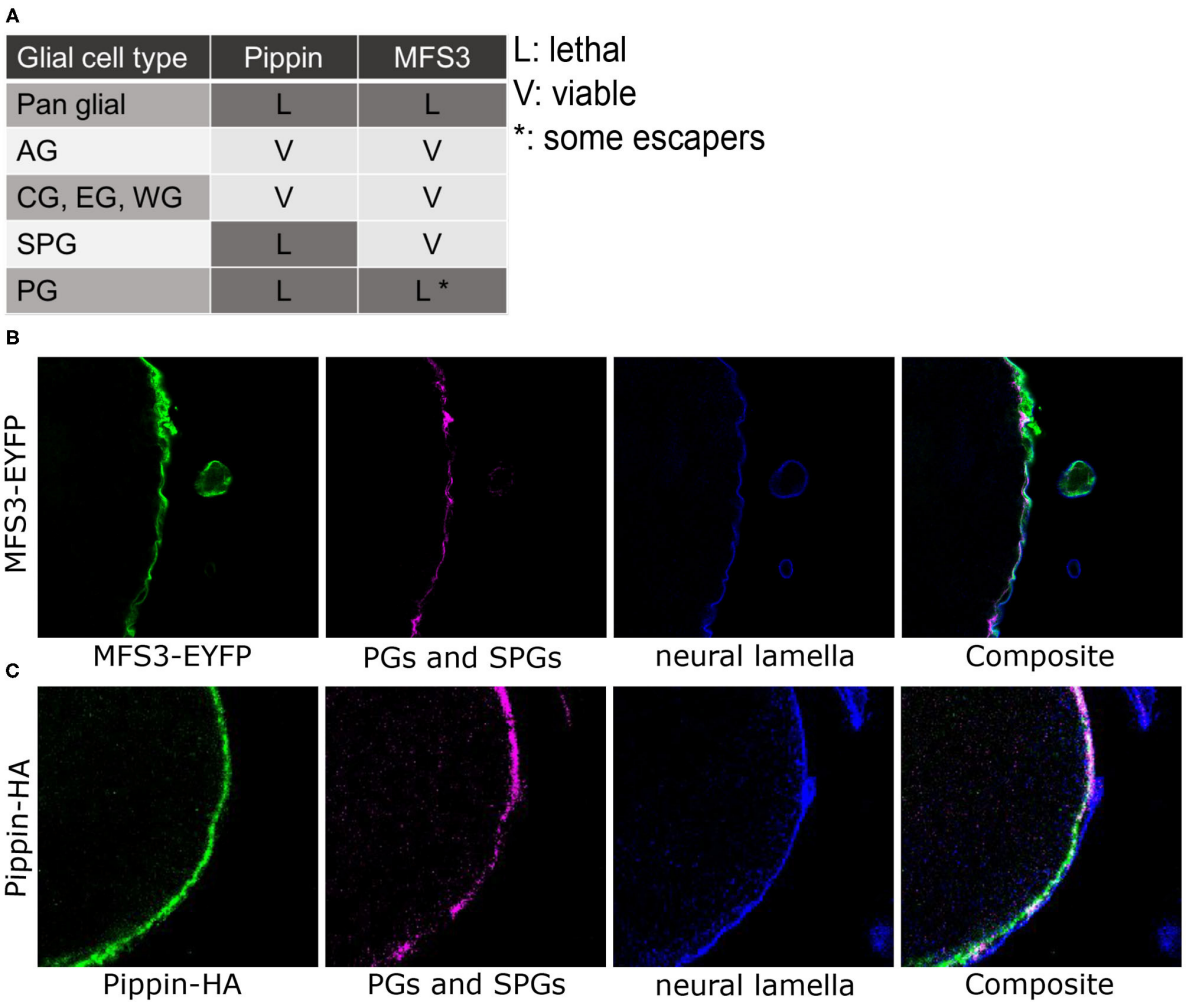
*CG4797* (*pippin*) and *Mfs3* encode putative BBB carbohydrate transporters. **(A)** RNAi interference-mediated silencing of *pippin* (using *CG4797*^*dsRNA10598*^) and *Mfs3 Mfs3*^*dsRNA4726R-3*^ in selected glial subtypes using glial subtype-specific Gal4 driver lines (pan glial: repo-Gal4; astrocyte-like glial cells (AG): alrm-Gal4; cortex glia (CG), ensheathing glia (EG), and wrapping glia (WG): nrv2-Gal4; subperineurial glial cells (SPG): Gli-Gal4, moody-Gal4; perineurial glial cells (PG): apt-Gal4, 46F-Gal4). Lethality was observed upon panglial and perineurial glia-specific suppression of *pippin* and *Mfs3*. In addition, lethality also occurred upon subperineurial glia-specific silencing of *pippin*. **(B)** Immunofluorescence staining of a transgenic *Pippin-HA* adult brain [green: Pippin-HA, magenta: NC120 (PGs and SPGs), blue: laminin (neural lamella)]. Pippin is found in subperineurial and perineurial glial cells. **(C)** Immunofluorescence staining of an MFS3-EYFP expressing adult brain [green: MFS3-EYFP, magenta: NC120 (PGs and SPGs), blue: laminin (neural lamella)]. MFS3-EYFP is expressed in the perineurial glial cells.

Previously, we showed that all cell types of the Drosophila nervous system are capable of taking up glucose (Volkenhoff et al., 2018). Since carbohydrates are hydrophilic molecules, they cannot diffuse over the plasma membrane and thus need to be transported. The only two carbohydrate transporters identified in the Drosophila CNS by now, Tret1-1 and Glut1, however, are expressed in the perineurial glial cells or the neurons, respectively. Thus, we set out to identify additional carbohydrate transporters expressed in the Drosophila nervous system. To this end we performed a small, biased RNA interference-based screen, in which we knocked down putative carbohydrate transporters encoded in the Drosophila genome specifically in the glial cells [genes with a predicted sugar transport function according to protein domain annotations from InterPro (http://www.ebi.ac.uk/interpro/) and UniProt (http://www.uniprot.org/), Supplementary Table 1]. This screen identified 14 putative carbohydrate transporters required in glial cells, amongst them CG4797 (Pippin) and Major Facilitator Superfamily Transporter 3 (MFS3, CG4762) (Supplementary Table 1). We focused our efforts on these two genes. Knockdown of the two genes specifically in glial cells using RNA interference (*pippin*^*dsRNA10598*^, *MFS3* ^*dsRNA4726R-3*^) leads to pupal lethality, indicating a function in glial cells (Figure 1A). Drosophila MFS3 shows 35% identity to the mouse anion/cation symporter (ACS) Sialin (NCBI protein blast), but the ACS consensus sequence is not fully conserved, indicating that MFS3 does not encode an ACS (Laridon et al., 2008). CG4797 encodes an SLC2 family glucose transporter most homologous to mouse GLUT6 and GLUT8 (NCBI protein blast). This indicates that *CG4797* encodes a carbohydrate transporter; thus, we named the gene *pippin*, after Frodo's friend, whose biggest concern is usually where to get the next meal.

To identify the glial subtype in which the putative transporters are needed, we repeated the knockdown experiments using glial subtype drivers (nrv2-Gal4: cortex glia, ensheathing glia and wrapping glia; alrm-Gal4: astrocyte-like glia cells; Gli-Gal4 or moody-Gal4: subperineurial glial cells; apt-Gal4 or 46F-Gal4: perineurial glial cells). Knockdown of *pippin* in perineurial or subperineurial glial cells led to lethality, while knockdown in any other glial subtype had no phenotypic consequences (Figure 1A). In contrast, knockdown of *Mfs3* only led to lethality in perineurial glial cells (Figure 1A). This indicates that Pippin is needed in both BBB-forming glial cells, while MFS3 is just essential in the perineurial glial cells. To verify the expression, we took advantage of an existing EYFP protein trap for MFS3 (MFS3^CPTI002305^). MFS3-EYFP localizes to the perineurial glial cells as seen when co-stained with NC120 (subperineurial glial cells) and laminin (neural lamella) (Figure 1B), as suggested from the knockdown experiments. To analyze the localization of *pippin*, we cloned the complete *pippin* locus, including upstream and downstream regions to include all regulatory elements, and fused a C-terminal 3xHA-tag to the coding sequence. Flies carrying this *pippin* minigene construct, show Pippin-HA expression in the perineurial and subperineurial glial cells as assumed from the RNAi-experiments (Figure 1C).

### Pippin and MFS3 Facilitate Carbohydrate Transport

**Figure 2:**
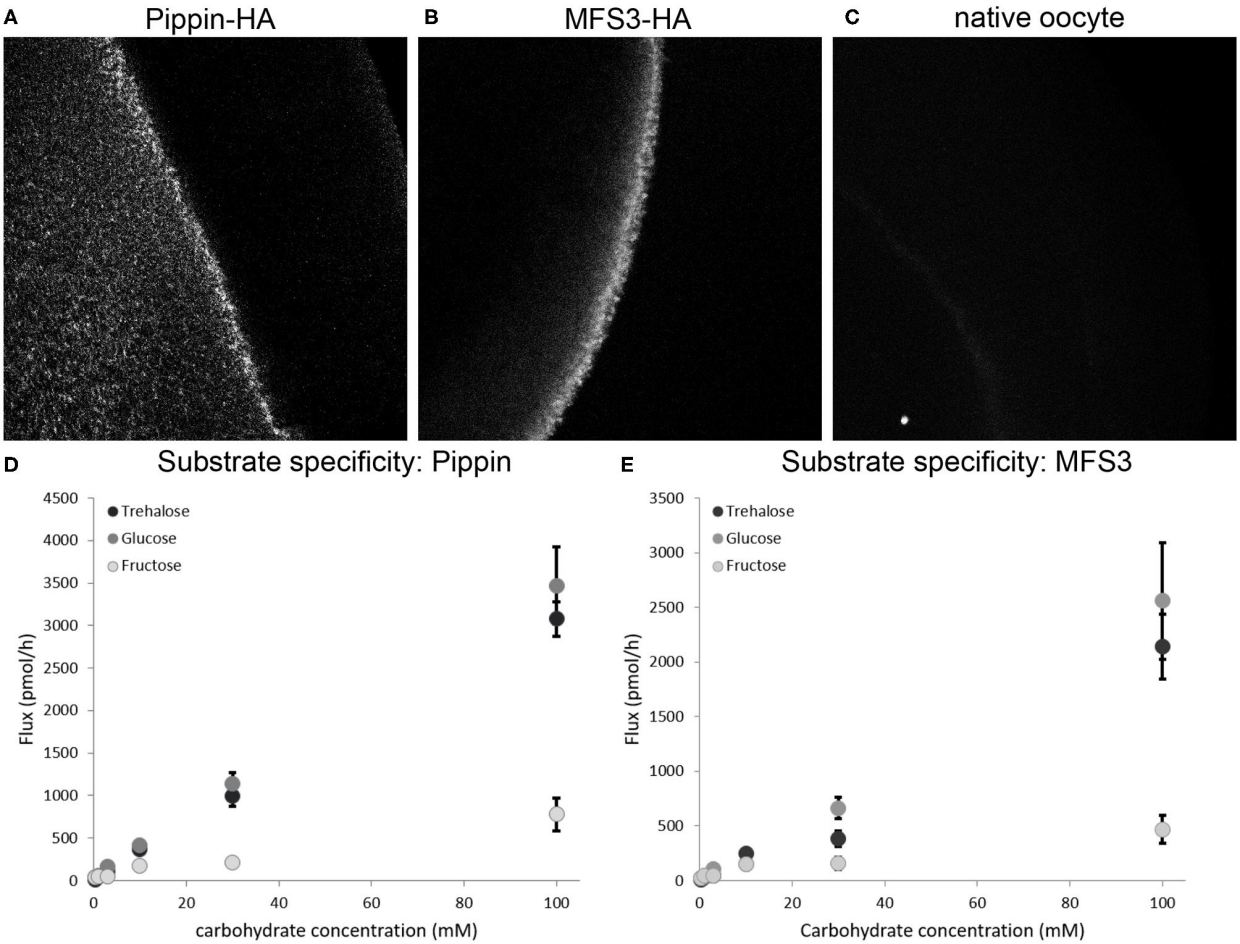
**(A,B)** Pippin and MFS3 facilitate carbohydrate transport. Heterologous expression of Pippin-HA and MFS3-HA in *Xenopus* oocytes **(A)** Pippin-HA **(B)** MFS3-HA are detectable on the surface of *Xenopus* oocytes (anti-HA immunostaining). **(C)** Native oocytes show no detectable HA staining. **(D,E)** Substrate specific uptake of ^14^C_12_-trehalose, ^14^C_6_-glucose and ^14^C_6_-fructose into *Xenopus* oocytes expressing **(D)** Pippin-HA or **(E)** MFS3-HA. Both Pippin and MFS3 show considerable transport capacity for trehalose and glucose, but not for fructose. Shown is the net-flux (flux observed in transporter-expressing oocytes minus flux observed in native oocytes). Values represent means ± standard error, *N* = 1–4.

To analyze whether the two newly identified BBB transporters are indeed able to facilitate carbohydrate uptake into the perineurial and/or subperineurial glial cells, we expressed Drosophila Pippin and MFS3 in *X. laevis* oocytes. To verify expression of the transporters we tagged Pippin and MFS3 with a 3xHA-tag. Both Pippin-HA and MFS3-HA are produced in Xenopus oocytes upon mRNA injection and localize to the membrane (Figures 2A–C). To analyze whether the transporters facilitate uptake of carbohydrates found in the Drosophila hemolymph, we incubated the respective oocytes with different concentrations of ^14^C-labeled glucose, trehalose, or fructose (Figures 2D,E). Interestingly, both Pippin and MFS3 facilitate uptake of glucose and trehalose efficiently (Figures 2D,E). Fructose, however, is transported at a much lower rate. Since naturally occurring fructose concentrations in the larva seem to be rather low compared to glucose and trehalose concentrations, it is unlikely that this transport is of physiological relevance (Mishra et al., 2013). These experiments show that the newly identified BBB transporters are indeed carbohydrate transporters. Fitting of the data, shown in Figures 2D,E, did not result in reliable K_m_ or V_max_ values. Therefore, more experiments need be carried out to analyze the transport kinetics of Pippin and MFS3 in Xenopus oocytes.

### Pippin and MFS3 Null Mutants Are Viable, but Display Shortened Lifespan and Reduced Locomotor Activity

To further analyze the consequences of loss of Pippin or MFS3, we generated null mutants for both transporters. We used CRISPR-mediated recombination to replace the entire coding sequence of *pippin* or *Mfs3* with a mini-white, thereby creating null mutants (Supplementary Figure 1). Interestingly, both *pippin*^−/−^ and *Mfs3*^−/−^ null mutants are viable and fertile, which contrasts with the phenotype observed upon glia-specific knockdown using RNA interference.

**Figure 3:**
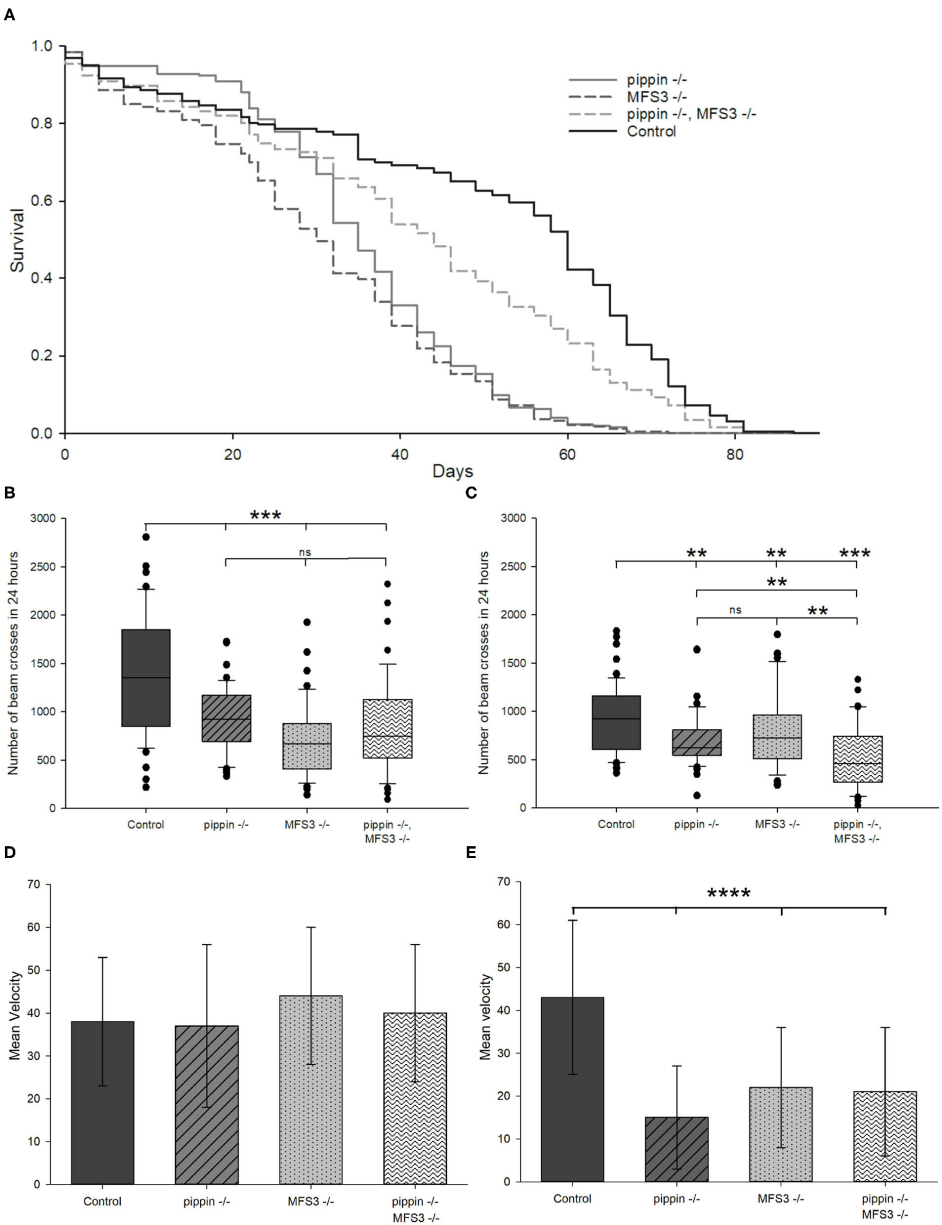
*pippin* and *Mfs3* null mutants are viable, but display shortened lifespan and reduced locomotor activity. **(A)** Survival curves of null mutants and control animals. All mutants show a reduced lifespan compared to control animals, however double *pippin*^−/−^, *Mfs3*^−/−^ mutants live significantly longer than single mutants. *N* = 3, *n* ≥ 180 (*pippin*^−/−^: *p* < 10^−21^, *Mfs3*^−/−^: *p* < 10^−23^, *pippin*^−/−^, *Mfs3*^−/−^: *p* < 10^−4^; log-rank test). **(B,C)** Activity monitored over 24 h of null mutants and control animals at 2 weeks old **(B)** and 5 weeks **(C)** of age. All mutants show a reduction in activity compared to control animals at 2 weeks of age, however the double mutants show a progressive reduction in activity moving significantly less than the single mutant at 5 weeks of age. *N* = 3; *n* ≥ 58; **p ≤ 0.01, ***p ≤ 0.001. **(D,E)** Negative geotaxis assay measuring climbing ability as an escape response of 2 **(D)** and 5 **(E)** weeks old flies. Single *pippin*^−/−^ or *Mfs3*^−/−^ mutants show no reduction in climbing ability at 2 weeks but show a decrease in activity at 5 weeks of age. *N* = 5; *n* ≥ 500; ****p ≤ 0.0001.

To assess viability of the mutants, we performed lifespan experiments. Indeed, *pippin*^−/−^ and *Mfs3*^−/−^ null mutants are short-lived compared to control animals (Figure 3A). Thus, we analyzed their phenotype in more detail. We assessed the activity of the null mutants after 2 and 5 weeks of age (Figures 3B,C). Already at the age of 2 weeks, both *pippin*^−/−^ and *Mfs3*^−/−^ null mutants are less active than control animals. To distinguish between a reduction in activity to save energy and the incapacity to move, we in addition studied the animals' escape response at the age of 2 and 5 weeks using a rapid iterative negative geotaxis (RING) assay (Gargano et al., 2005), in which the flies are put in vials that are tapped on the table. This tapping induces an escape response, where the flies run up the walls of the vial. Depending on their locomotor capabilities the animals climb the walls faster or slower (Figures 3D,E). At the age of 2 weeks all genotypes are capable of a fast escape response (Figure 3D). Thus, at this age, the animals are able to move as well as control flies, but are nevertheless less active, most likely as a means of saving energy. However, at the age of 5 weeks the velocity of *pippin*^−/−^ and *Mfs3*^−/−^ null mutants is significantly reduced, indicating progressive loss of locomotor abilities (Figure 3E).

Since RNAi-mediated knockdown of *pippin* or *Mfs3* are pupal lethal, but the null mutants are not, we checked for putative compensation of the loss of either transporter. To this end, we created double *pippin*^−/−^, *Mfs3*^−/−^ mutants and analyzed their phenotype. Interestingly, *pippin*^−/−^, *Mfs3*^−/−^ double mutants are also viable and fertile. Surprisingly, lifespan experiments show that the double mutants live longer than the respective single mutants, albeit not as long as control animals (Figure 3A). To establish whether the double mutants move even less than the single mutants to save energy, we analyzed their activity at 2 and 5 weeks of age (Figures 3B,C). At 2 weeks of age the double mutant is significantly less active than wildtype control animals but moves as much as either single mutant (Figure 3B). In contrast, at the age of 5 weeks, the double mutant animals are significantly less active than either single mutant (Figure 3C). To distinguish between an inability to move and an energy-saving reduction of activity, we also analyzed the escape response. Here, the double mutant animals are indistinguishable from single mutant animals at either time point (Figures 3D,E). This indicates that the double mutant animals have the ability to move as well as the single mutants. However, they seem to move progressively less over their lifespan, probably to save energy.

### Compensatory Increase in Circulating Carbohydrate Levels and Upregulation of Tret1-1 Upon Loss of Pippin or MFS3

**Figure 4:**
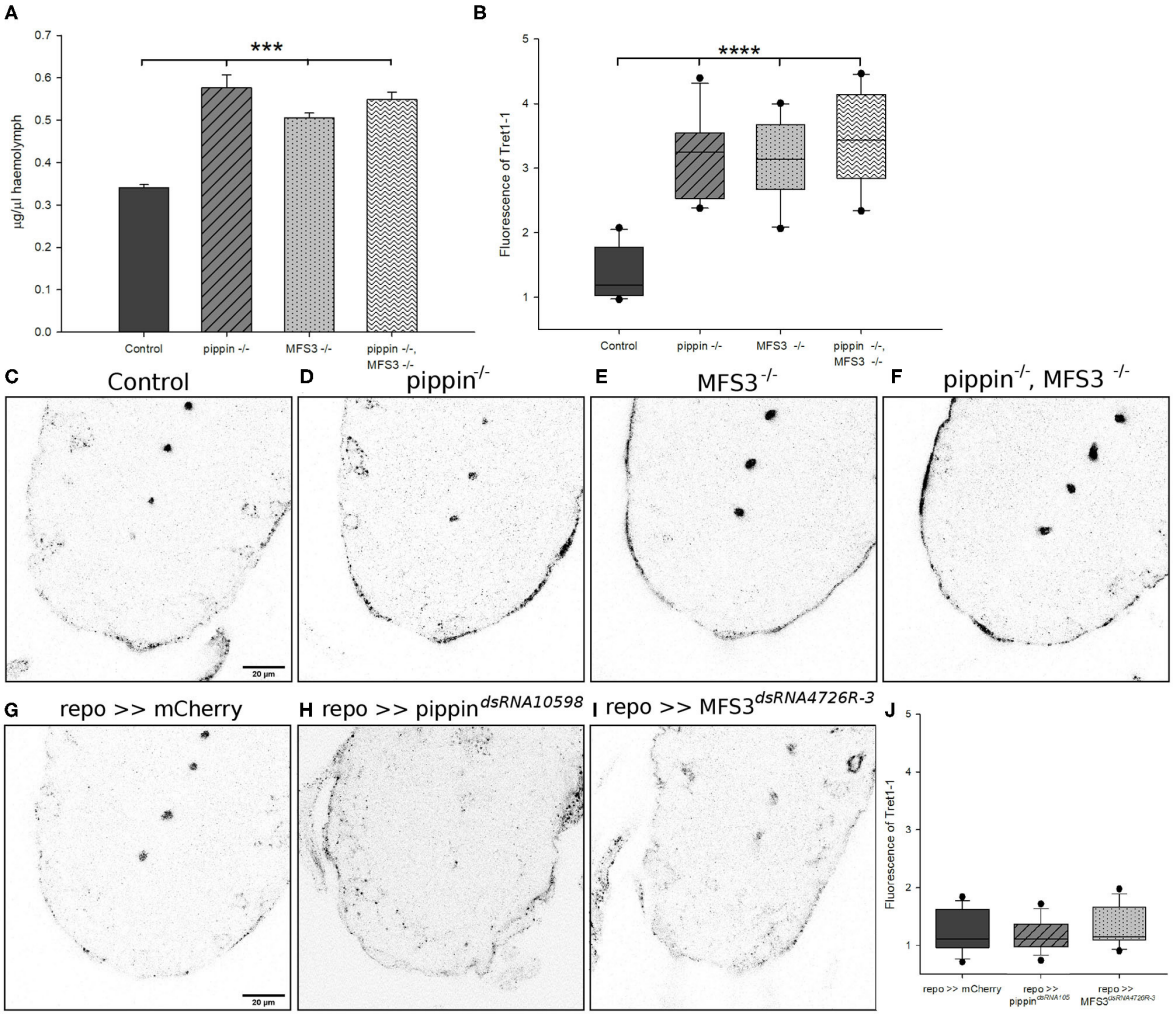
Compensatory increase in circulating carbohydrate levels and upregulation of Tret1-1 upon loss of Pippin or MFS3. **(A)** Glucose levels in the hemolymph of adult flies were analyzed. Both single and double *pippin*^−/−^ and *Mfs3*^−/−^ mutants show an increase in circulating glucose levels. *N* = 3; ***p ≤ 0.001. **(B–J)** L3 larval brains of null mutants or panglial knockdown of *pippin* and *Mfs3* (*repo-Gal4*> *CG4797*^*dsRNA10598*^; *repo-Gal4*>*MFS3*^*dsRNA4726R-3*^) were dissected and stained for Tret1-1 expression. **(B)** Quantification shows the difference in Tret1-1 fluorescence between controls and null mutants. *N* ≥ 4; *n* = 10–12; ****p ≤ 0.0001. **(C)** Control animals show wildtype levels of Tret1-1 in the brain. **(D)** *pippin*^−/−^ mutants **(E)**, *Mfs3*^−/−^ mutants **(F)**, and *pippin*^−/−^, *Mfs3*^−/−^ double mutants show a strong upregulation in Tret1-1 expression in perineurial glial cells. **(G)** *repo*>>*mCherry*^*dsRNA*^ serves as a control for panglial knockdown of *pippin* and *Mfs3* by RNA interference. *repo*>>*mCherry*^*dsRNA*^ animals show wildtypic levels of Tret1-1. **(H)** Glia-specific knockdown of *pippin* (*repo*>>*CG4797*^*dsRNA10598*^). No increase in Tret1-1 levels can be found. **(I)** Glia-specific knockdown of *Mfs3* (*repo*>>*MFS3*^*dsRNA4726R-3*^). No increase in Tret1-1 levels can be seen. **(J)** Quantification of Tret1-1 fluorescence of pan glial knockdown of mCherry, *pippin* or *Mfs3*. *N* ≥ 4; *n* = 14–15.

To understand why *pippin*^−/−^ and *Mfs3*^−/−^ null mutants are viable, while glia-specific acute knockdown is lethal, we investigated other possible compensatory mechanisms. Classic carbohydrate transporters, like SLC2 family carbohydrate transporters, are facilitative transporters, which means that they allow uptake of the respective carbohydrate into a cell driven by a concentration gradient. Thus, an increase in the concentration gradient between the extracellular milieu and the cytosol of the respective cell, accelerates carbohydrate uptake into the cell. Therefore, we analyzed circulating glucose levels in *pippin*^−/−^ and *Mfs3*^−/−^ null mutants to see if deficits in transporter expression might be compensated by elevated circulating sugar levels (Figure 4A). Indeed, *pippin*^−/−^ and *Mfs3*^−/−^ null mutants, as well as the double mutants display elevated hemolymph glucose levels that might facilitate glucose uptake into the brain.

An alternative mode of compensation for the loss of a carbohydrate transporter would be to upregulate an alternative transporter. The only other carbohydrate transporter known to be expressed in the Drosophila BBB, besides Pippin and MFS3, is Tret1-1 (Volkenhoff et al., 2015). As Pippin and MFS3, Tret1-1 facilitates uptake of glucose and trehalose when heterologously expressed in Xenopus oocytes (Kanamori et al., 2010; Hertenstein et al., 2020). To assess if Tret1-1 could compensate for the loss of either Pippin or MFS3 in the null mutants, we stained null mutant L3 brains for Tret1-1 expression in the perineurial glial cells. Interestingly, Tret1-1 expression is strongly increased in the perineurial glial cells of *pippin*^−/−^ and *Mfs3*^−/−^ null mutants as well as *pippin*^−/−^, *Mfs3*^−/−^ double mutants (Figures 4B–F). The increase in Tret1-1 expression is not significantly higher in the double mutants than in the single mutants. Loss of Pippin, however, does not induce compensatory misexpression of Tret1-1 in the subperineurial glial cells (Supplementary Figure 2). This increase in Tret1-1 expression in the perineurial glial cells could compensate for a reduction of carbohydrate uptake caused by loss of Pippin and/or MFS3. To understand the difference between RNAi-mediated knockdown of *pippin* and *Mfs3* and the null mutants, we also analyzed Tret1-1 expression in animals with a glia-specific knockdown of either *pippin* or *Mfs3* (Figures 4G–J). Indeed, glia-specific knockdown of *pippin* or *Mfs3* does not induce a compensatory upregulation of Tret1-1, potentially explaining the phenotypic differences (Figures 4G–J). These findings suggest that null mutations, like a complete loss of the coding region as in the case of our *pippin*^−/−^ and *Mfs3*^−/−^ mutants, induce different compensatory mechanisms than constant degradation of the respective mRNAs, as induced by RNA interference. If such differences in compensation are common, this could explain the discrepancies often found between RNAi-mediated knockdown phenotypes and null mutant phenotypes.

### Pippin and MFS3 Facilitate Glucose Uptake in the Drosophila BBB

**Figure 5:**
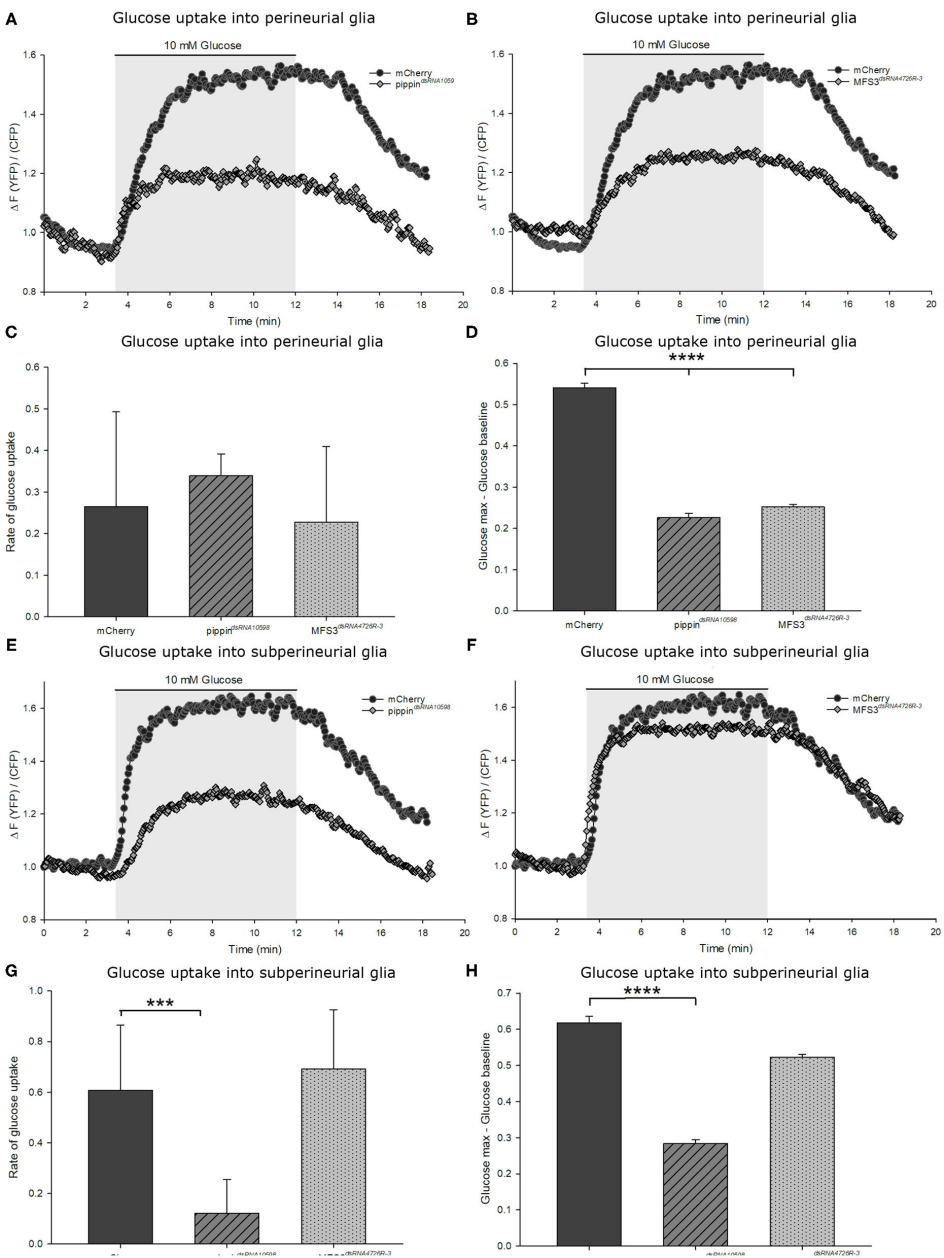
Pippin and MFS3 facilitate glucose uptake into the Drosophila BBB-forming cells. Glucose uptake was measure in *ex vivo* L3 larval brains expressing a genetically encoded glucose sensor (FLII^12^Pglu-700μδ6). **(A,B)** Example traces of brains with a perineurial knockdown of *pippin* (*apt-Gal4*>*pippin*^*dsRNA10598*^) **(A)** or *Mfs3* (*apt-Gal4*>*MFS3*^*dsRNA4726R-3*^) **(B)**, where the glucose sensor is expressed in the perineural glial cells. **(C)** Quantification of the rate of glucose uptake. The glucose uptake rate as calculated by the steepness of the slope shows no difference between transporter knockdown and control brains. **(D)** Quantification of the maximum glucose concentration in the cells. Shown is the difference between the maximum glucose concentration and the baseline glucose concentration. *N* = 8–12; ****p ≤ 0.0001. **(E–H)** Glucose uptake into the subperineurial glial cells. **(E)** *moody-gal4*>*pippin*^*dsRNA10598*^ brains show a reduction in the uptake rate and overall levels of glucose entering the subperineurial glia. **(F)** *moody-Gal4*>*Mfs3*^*dsRNA4726R-3*^ brains show no difference in glucose uptake rate or maximum glucose levels. **(G,H)** Quantification of the glucose uptake rate and the maximum glucose concentrations reached in the subperineurial glial cells. **(G)** Rate of glucose uptake into the subperineurial glial cells in brains of the different genotypes (subperineurial glial knockdown). Knockdown of *pippin* in the subperineurial glial cells severely reduces glucose uptake rates. **(H)** Maximum glucose levels in subperineurial glial cells expressing FLII^12^Pglu-700μδ6. Brains in which *pippin* is knocked down in the subperineurial glial cells show a lower maximum glucose level than control brains or *Mfs3* knockdown brains.^.^*n* = 8–12; ***p ≤ 0.001, ****p ≤ 0.0001. Error bars show standard deviation.

**Figure 6:**
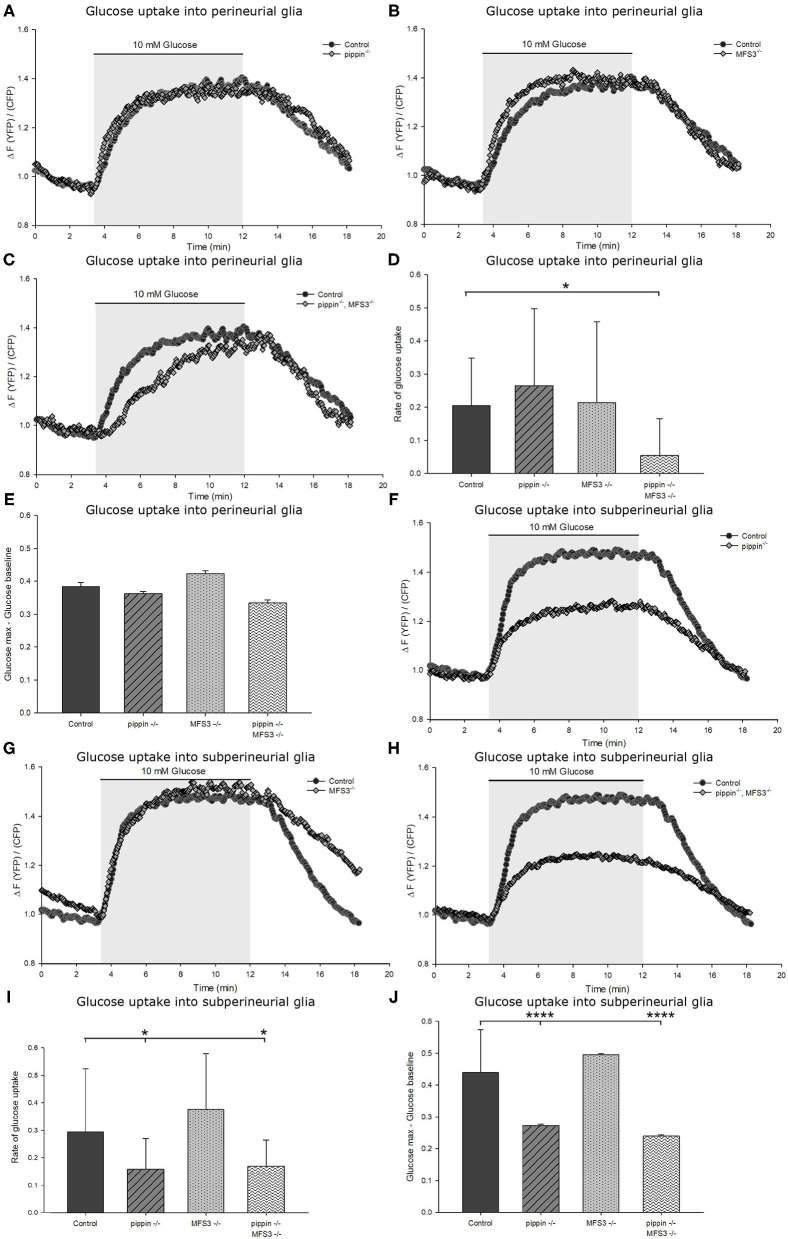
Compensatory upregulation rescues deficits in carbohydrate uptake caused by loss of Pippin and MFS3**. (A–E)** Capacity of glucose uptake in *ex vivo* larval brains of *pippin*^−/−^ or *Mfs3*^−/−^ null single or double mutants expressing FLII^12^Pglu-700μδ6 in the perineurial glial cells. There is no difference observed in glucose uptake rate or maximum glucose concentration compared to control brains for *pippin*^−/−^ **(A)** or *Mfs3*^−/−^ **(B)** single mutants. However, the double mutant **(C)** perineurial glial cells take up glucose significantly slower than single mutants or wildtype controls. **(D)** Quantification of the glucose uptake rate into perineurial glial cells in the indicated genotypes. The double mutant perineurial glial cells take up glucose significantly slower than single mutants or wildtype controls. *n* = 8–12; *p ≤ 0.05. **(E)** Quantification of the difference between maximum glucose concentration and baseline glucose concentration. There is no observable difference between the genotypes. *n* = 8–12. **(F–J)** Glucose uptake and maximum glucose level in the subperineurial glial cells. **(F)** *pippin*^−/−^ mutant brains, but not *Mfs3*^−/−^ mutant brains **(G)** show reduced glucose uptake into the subperineurial glial cells. **(H)** *pippin*^−/−^*, MFS3*^−/−^ double mutant brains show the same phenotype as *pippin*^−/−^ single mutant brains. Both the rate of glucose uptake **(I)** and the maximum glucose concentration **(J)** are reduced in both *pippin*^−/−^ single and *pippin*^−/−^*, MFS3*^−/−^ double mutant brains. *n* = 8–12; *p ≤ 0.05, ****p ≤ 0.0001. Error bars show standard deviation.

To study if loss of any of the described carbohydrate transporters has an effect on carbohydrate uptake into the BBB-forming glial cells, we analyzed glucose uptake into the respective cells using a genetically-encoded Förster resonance energy transfer (FRET)-based glucose sensor, FLII12Pglu-700μδ6 (Fehr et al., 2003; Takanaga et al., 2008; Volkenhoff et al., 2018). This sensor allows visualizing carbohydrate uptake in living *ex vivo* L3 larval brains (Volkenhoff et al., 2018). To understand the changes in carbohydrate uptake in the different mutants and knockdown animals, we expressed the glucose sensor either in the perineurial or subperineurial glial cells of the animals and analyzed glucose uptake capacity (Figures 5, 6). When we analyzed glucose uptake into the perineurial glial cells of animals with a perineurial glia-specific knockdown of *pippin* (using apt-Gal4), we found that the maximum concentration of glucose found in the cells is significantly reduced compared to that found in control animals (expressing mCherry-dsRNA) (Figures 5A,D). This indicates that Pippin indeed acts as a carbohydrate transporter in the perineurial glial cells and that loss of Pippin reduces glucose uptake efficiency significantly. Interestingly, the initial glucose uptake rate does not change (Figure 5C). Since Pippin is also expressed in the subperineurial glial cells, we also analyzed glucose uptake into those cells. In this case, we expressed the dsRNA-construct as well as the glucose sensor using moody-Gal4. As expected upon loss of a carbohydrate transporter, both the glucose uptake rate as well as the maximal glucose concentration reached in the cells are significantly decreased upon loss of Pippin in the subperineurial glial cells (Figures 5E–H). Upon RNAi-mediated loss of MFS3, glucose transport is impaired in the perineurial glial cells, but not in the subperineurial glial cells (Figures 5B–D,F–H). This fits the expression of MFS3 in the perineurial but not in the subperineurial glial cells and suggests that, indeed, also MFS3 is essential for glucose transport into the perineurial glial cells.

### Compensatory Upregulation Rescues Deficits in Carbohydrate Uptake Caused by Loss of Pippin and MFS3

To analyze if compensatory upregulation of Tret1-1 can rescue glucose uptake efficiency in the Drosophila BBB, we analyze glucose uptake into the perineurial and subperineurial glial cells in *pippin*^−/−^ and *Mfs3*^−/−^ null mutant animals. *pippin*^−/−^ and *Mfs3*^−/−^ null mutant animals display wild typic glucose uptake into the perineurial glial cells (Figures 6A–D). Thus, compensatory upregulation of Tret1-1 indeed rescues deficits in carbohydrate transport. As expected, glucose uptake into the subperineurial glial cells is indistinguishable from controls in *Mfs3*^−/−^ null mutant animals (Figure 6G). In contrast, *pippin*^−/−^ mutant animals show reduced glucose uptake efficiency into the subperineurial glial cells, indicating a lack of compensation in this cell type (Figures 6F–J). These results match the expectations, since MFS3 is not expressed in the subperineurial glial cells and subperineurial loss of Pippin is not compensated for by Tret1-1 upregulation.

We also analyzed carbohydrate uptake into the perineurial and subperineurial glial cells of *pippin*^−/−^, *Mfs3*^−/−^ double mutants. Here, we find reduced uptake efficiency in both the perineurial and subperineurial glial cells (Figures 6C,D,H–J). The reduction of glucose uptake into the subperineurial glial cells most likely phenocopies the reduction found in *pippin*^−/−^ null mutants, since Pippin is the only transporter expressed in those cells. Interestingly, Tret1-1 upregulation does not seem to be sufficient to rescue glucose transport deficits caused by loss of both Pippin and MFS3 in the perineurial glial cells (Figures 6C,D). This finding might explain the differences in lifespan and activity between the single and the double mutant animals.

## Discussion

Sufficient nutrient supply to the nervous system is essential for its proper function. Since the main energy source of the brain is sugar, adequate carbohydrate transport over the BBB needs to be ensured. Thus, the vertebrate as well as the insect BBB-forming cells express carbohydrate transporters to facilitate uptake of sugars (Weiler et al., 2017). We report the identification of two additional carbohydrate transporters expressed by the BBB-forming glial cells of Drosophila, Pippin, and MFS3. Both transporters can facilitate uptake of glucose and trehalose (Figure 2). RNAi-mediated knockdown of either gene induces pupal lethality, while null mutants are viable and fertile. This discrepancy is found since null mutants show a compensatory upregulation of the carbohydrate transporter Tret1-1. Interestingly, such upregulation cannot be seen in knockdown animals, suggesting that there is a major difference in compensation if the mRNA of a certain gene is produced and then degraded or if there is no mRNA production since the coding sequence has been deleted. Similar discrepancies have been found comparing morpholino-induced knockdown phenotypes vs. mutant phenotypes in zebrafish or siRNA-mediated knockdown phenotypes vs. mutant phenotypes in mice (De Souza et al., 2006; Daude and Westaway, 2012; Kok et al., 2015; Rossi et al., 2015). In zebrafish, for example, Egfl7 null mutants show compensatory upregulation of Emilin genes that rescue Egfl7 loss. Such upregulation is not found in morpholino-knockdowns that thus show a severe vascular defects (Rossi et al., 2015). Interestingly, such compensation might even be conserved in humans. On Iceland individuals with a homozygous loss of Egfl7 were identified, who do not suffer from any symptoms (Sulem et al., 2015). However, the underlying regulatory mechanisms are currently unknown. They are likely to be complex and will probably require much effort to unravel. In any case, such conserved differential compensation should be considered when studying the effects of gene knockdown and null mutations.

The data reported here shows that transporter expression at the BBB can be adapted to suboptimal circumstances, like in this case loss of one transporter. There are two potential mechanisms that could compensate for transporter loss: increase of the concentration gradient at the plasma membrane (circulation vs. cytosol), and compensatory upregulation of another transporter. In case of our null mutant flies we see compensation via both possibilities (Figure 4). The animals display higher circulating sugar concentrations that most likely increase the concentration gradient over the plasma membrane and thus make carbohydrate transport via facilitative transporters more efficient, as well as an upregulation of another transporter, Tret1-1. These compensatory mechanisms rescue transport efficiency as seen using a genetically encoded glucose sensor to assess glucose uptake properties (Figure 6). The increase in circulating carbohydrates suggests a crosstalk between the nervous system, probably the BBB-forming glial cells, and the periphery to regulate nutrient mobilization most likely form the fat body. That BBB transport defects can regulate systemic metabolism is a very interesting finding that will foster exciting follow up studies to unravel the regulatory mechanisms.

It has been shown previously that Tret1-1 is upregulated upon starvation-induced hypoglycemia (Hertenstein et al., 2020). Together with the data reported here, this suggests that any alteration that leads to insufficient carbohydrate uptake results in compensatory upregulation of transport proteins, most likely to ensure sufficient energy provision to the nervous system. In the case of starvation, Tret1-1 is upregulated via TGF-β signaling (Hertenstein et al., 2020). Since this signaling seems to be induced by hypoglycemia, it is very unlikely that TGF-β signaling is also regulating compensatory upregulation in the case of transporter loss (Hertenstein et al., 2020). Mammalian GLUT1 and SGLT1 and 2 have also been shown to be dynamically upregulated upon hypoglycemia or other insults like oxygen and glucose deprivation as a result of ischemia (Boado and Pardridge, 1993; Kumagai et al., 1995; Nishizaki et al., 1995; Nishizaki and Matsuoka, 1998; Simpson et al., 1999, reviewed in Elfeber et al., 2004; Enerson and Drewes, 2006; Vemula et al., 2009; Yu et al., 2013; Patching, 2016; Rehni and Dave, 2018). Thus, it is very likely that a flexible and dynamic regulation of carbohydrate transporters is an evolutionary conserved mechanism that ensures proper nervous system function even under suboptimal conditions. Since aberrations in carbohydrate availability and transport are thought to be a major cause of severe illnesses, like GLUT1 deficiency syndrome, Alzheimer's disease or epilepsy (Kapogiannis and Mattson, 2011; Arsov et al., 2012; Hoffmann et al., 2013; Koepsell, 2020), it will be very interesting to unravel the regulatory mechanisms that can lead to a compensation of insufficient carbohydrate uptake. Studying these mechanisms might enable us in the future to treat the effects of insufficient carbohydrate uptake at the BBB.

## Data Availability Statement

The raw data supporting the conclusions of this article will be made available by the authors, without undue reservation.

## Ethics Statement

The animal study was reviewed and approved by the Landesuntersuchungsamt Rheinland-Pfalz, Koblenz (23 177-07/A07-2-003 §6).

## Author Contributions

EM designed and conducted most experiments, helped conceiving the study, and wrote the paper with SS. AW conducted the Xenopus experiments together with HB and did some of the fly experiments. HB designed the Xenopus experiments and helped conducting them. SS conceived the study, assisted in designing and interpreting experiments, and wrote the paper with EM and obtained funding from the DFG. All authors contributed to the article and approved the submitted version.

## Conflict of Interest

The authors declare that the research was conducted in the absence of any commercial or financial relationships that could be construed as a potential conflict of interest.

## References

[B1] Arsov T. Mullen S. A. Damiano J. A. Lawrence K. M Huh L. L. Nolan M.. (2012). Early onset absence epilepsy: 1 in 10 cases is caused by GLUT1 deficiency. Epilepsia 53, e204–e207. 10.1111/epi.12007 23106342

[B2] Becker H. M. (2014). Transport of lactate: characterization of the transporters involved in transport at the plasma membrane by heterologous protein expression in xenopus oocytes, in Neuromethods, eds Hirrlinger J. Waagepetersen H. (New York, NY: Springer), 25–43.

[B3] Becker H. M. Bröer S. Deitmer J. W. (2004). Facilitated lactate transport by MCT1 when coexpressed with the sodium bicarbonate cotransporter (NBC) in xenopus oocytes. Biophys. J. 86, 235–247. 10.1016/S0006-3495(04)74099-0 14695265 PMC1303786

[B4] Birnbaum M. J. Haspel H. C. Rosen O. M. (1986). Cloning and characterization of a cDNA encoding the rat brain glucose-transporter protein. Proc. Natl. Acad. Sci. U.S.A. 83, 5784–5788. 10.1073/pnas.83.16.5784 3016720 PMC386379

[B5] Boado R. J. Pardridge W. M. (1993). Glucose deprivation causes posttranscriptional enhancement of brain capillary endothelial glucose transporter gene expression via GLUT1 mRNA stabilization. J. Neurochem. 60, 2290–2296. 10.1111/j.1471-4159.1993.tb03516.x 8098356

[B6] Broughton S. Alic N. Slack C. Bass T. Ikeya T. Vinti G.. (2008). Reduction of DILP2 in Drosophila triages a metabolic phenotype from lifespan revealing redundancy and compensation among DILPs. PLoS ONE 3:e3721. 10.1371/journal.pone.0003721 19005568 PMC2579582

[B7] Croset V. Treiber C. D. Waddell S. (2018). Cellular diversity in the Drosophila midbrain revealed by single-cell transcriptomics. Elife 7:e34550. 10.7554/eLife.34550 29671739 PMC5927767

[B8] Daude N. Westaway D. (2012). Shadoo/PrP (Sprn0/0/Prnp0/0) double knockout mice: more than zeroes. Prion 6, 420–424. 10.4161/pri.21867 22929230 PMC3510864

[B9] Davie K. Janssens J. Koldere D. De Waegeneer M. Pech U. Kreft Ł.. (2018). A single-cell transcriptome atlas of the aging drosophila brain. Cell 174, 982–998.e20. 10.1016/j.cell.2018.05.057 29909982 PMC6086935

[B10] De Souza A. T. Dai X. Spencer A. G. Reppen T. Menzie A. Roesch P. L.. (2006). Transcriptional and phenotypic comparisons of Ppara knockout and siRNA knockdown mice. Nucleic Acids Res. 34, 4486–4494. 10.1093/nar/gkl609 16945951 PMC1636368

[B11] Desalvo M. K. Hindle S. J. Rusan Z. M. Orng S. Eddison M. Halliwill K.. (2014). The Drosophila surface glia transcriptome: evolutionary conserved blood-brain barrier processes. Front. Neurosci. 8:346. 10.3389/fnins.2014.00346 25426014 PMC4224204

[B12] Dick A. P. Harik S. I. Klip A. Walker D. M. (1984). Identification and characterization of the glucose transporter of the blood-brain barrier by cytochalasin B binding and immunological reactivity. Proc. Natl. Acad. Sci. U.S.A. 81, 7233–7237. 6150484 10.1073/pnas.81.22.7233 PMC392113

[B13] Elfeber K. Köhler A. Lutzenburg M. Osswald C. Galla H. J. Witte O. W.. (2004). Localization of the Na+-D-glucose cotransporter SGLT1 in the blood-brain barrier. Histochem. Cell Biol. 121, 201–207. 10.1007/s00418-004-0633-9 14986005

[B14] Enerson B. E. Drewes L. R. (2006). The rat blood-brain barrier transcriptome. J. Cereb. Blood Flow Metab. 26, 959–973. 10.1038/sj.jcbfm.9600249 16306934

[B15] Engler C. Kandzia R. Marillonnet S. (2008). A one pot, one step, precision cloning method with high throughput capability. PLoS ONE 3:e3647. 10.1371/journal.pone.0003647 18985154 PMC2574415

[B16] Farrell C. L. Pardridge W. M. (1991). Blood-brain barrier glucose transporter is asymmetrically distributed on brain capillary endothelial lumenal and ablumenal membranes: an electron microscopic immunogold study. Proc. Natl. Acad. Sci. U.S.A. 88, 5779–5783. 2062858 10.1073/pnas.88.13.5779 PMC51961

[B17] Fehr M. Lalonde S. Lager I. Wolff M. W. Frommer W. B. (2003). *In vivo* imaging of the dynamics of glucose uptake in the cytosol of COS-7 cells by fluorescent nanosensors. J. Biol. Chem. 278, 19127–19133. 10.1074/jbc.M301333200 12649277

[B18] Gargano J. W. Martin I. Bhandari P. Grotewiel M. S. (2005). Rapid iterative negative geotaxis (RING): a new method for assessing age-related locomotor decline in Drosophila. Exp. Gerontol. 40, 386–395. 10.1016/j.exger.2005.02.005 15919590

[B19] Gerhart D. Z. Levasseur R. J. Broderius M. A. Drewes L. R. (1989). Glucose transporter localization in brain using light and electron immunocytochemistry. J. Neurosci. Res. 22, 464–472. 10.1002/jnr.490220413 2668543

[B20] Harik S. I. Kalaria R. N. Andersson L. Lundahl P. Perry G. (1990). Immunocytochemical localization of the erythroid glucose transporter: abundance in tissues with barrier functions. J. Neurosci. 10, 3862–3872. 2269888 10.1523/JNEUROSCI.10-12-03862.1990 PMC6570044

[B21] Harris J. J. Jolivet R. Attwell D. (2012). Synaptic energy use and supply. Neuron 75, 762–777. 10.1016/j.neuron.2012.08.019 22958818

[B22] Hertenstein H. McMullen E. Weiler A. Volkenhoff A. Becker H. M. Schirmeier S. (2020). Starvation-induced regulation of carbohydrate transport at the blood-brain barrier is TGF-β-signaling dependent. bioRxiv. 10.1101/2020.09.21.306308 PMC8149124 34032568

[B23] Hindle S. J. Bainton R. J. (2014). Barrier mechanisms in the Drosophila blood-brain barrier. Front. Neurosci. 8:414. 10.3389/fnins.2014.00414 25565944 PMC4267209

[B24] Hoffmann U. Sukhotinsky I. Eikermann-Haerter K. Ayata C. (2013). Glucose modulation of spreading depression susceptibility. J. Cereb. Blood Flow Metab. 33, 191–195. 10.1038/jcbfm.2012.132 22968322 PMC3564186

[B25] Hummel T. Attix S. Gunning D. Zipursky S. L. (2002). Temporal control of glial cell migration in the Drosophila eye requires gilgamesh, hedgehog, and eye specification genes. Neuron. 33, 193–203. 10.1016/S0896-6273(01)00581-5 11804568

[B26] Kanamori Y. Saito A. Hagiwara-Komoda Y. Tanaka D. Mitsumasu K. Kikuta S.. (2010). The trehalose transporter 1 gene sequence is conserved in insects and encodes proteins with different kinetic properties involved in trehalose import into peripheral tissues. Insect Biochem. Mol. Biol. 40, 30–37. 10.1016/j.ibmb.2009.12.006 20035867

[B27] Kapogiannis D. Mattson M. P. (2011). Disrupted energy metabolism and neuronal circuit dysfunction in cognitive impairment and Alzheimer's disease. Lancet. Neurol. 10, 187–198. 10.1016/S1474-4422(10)70277-5 21147038 PMC3026092

[B28] Koehn L. (2020). ABC efflux transporters at blood-central nervous system barriers and their implications for treating spinal cord disorders. Neural Regen. Res. 15, 1235–1242. 10.4103/1673-5374.272568 31960802 PMC7047801

[B29] Koepsell H. (2020). Glucose transporters in brain in health and disease. Pflügers Arch. Eur. J. Physiol. 472, 1299–1343. 10.1007/s00424-020-02441-x 32789766 PMC7462931

[B30] Kok F. O. Shin M. Ni C. W. Gupta A. Grosse A. S. VanImpel A.. (2015). Reverse genetic screening reveals poor correlation between morpholino-induced and mutant phenotypes in zebrafish. Dev. Cell. 32, 97–108. 10.1016/j.devcel.2014.11.018 25533206 PMC4487878

[B31] Kumagai A. K. Kang Y.-S. Boado R. J. Pardridge W. M. (1995). Upregulation of blood-brain barrier GLUT1 glucose transporter protein and mRNA in experimental chronic hypoglycemia. Diabetes 44, 1399–1404. 10.2337/diab.44.12.1399 7589845

[B32] Kuzawa C. W. Chugani H. T. Grossman L. I. Lipovich L Muzik O. Hof P. R.. (2014). Metabolic costs and evolutionary implications of human brain development. Proc. Natl. Acad. Sci. U.S.A. 111, 13010–13015. 10.1073/pnas.1323099111 25157149 PMC4246958

[B33] Laridon B. Callaerts P. Norga K. (2008). Embryonic expression patterns of Drosophila ACS family genes related to the human sialin gene. Gene Expr. Patterns 8, 275–283. 10.1016/j.gep.2007.12.003 18255354

[B34] Laughlin S. B. de Ruyter Van Steveninck R. R. Anderson J. C. (1998). The metabolic cost of neural information. Nat. Neurosci. 1, 36–41. 10.1038/236 10195106

[B35] Lee G. Park J. H. (2004). Hemolymph sugar homeostasis and starvation-induced hyperactivity affected by genetic manipulations of the adipokinetic hormone-encoding gene in *Drosophila melanogaster*. Genetics 167, 311–323. 10.1534/genetics.167.1.311 15166157 PMC1470856

[B36] Limmer S. Weiler A. Volkenhoff A. Babatz F. Klämbt C. (2014). The Drosophila blood-brain barrier: development and function of a glial endothelium. Front. Neurosci. 8:365. 10.3389/fnins.2014.00365 25452710 PMC4231875

[B37] Löscher W. Potschka H. (2005). Blood-brain barrier active efflux transporters: ATP-binding cassette gene family. NeuroRx 2, 86–98. 10.1602/neurorx.2.1.86 15717060 PMC539326

[B38] Maher F. Davies-Hill T. M. Lysko P. G. Henneberry R. C. Simpson I. A. (1991). Expression of two glucose transporters, GLUT1 and GLUT3, in cultured cerebellar neurons: evidence for neuron-specific expression of GLUT3. Mol. Cell. Neurosci. 2, 351–360. 10.1016/1044-7431(91)90066-W 19912819

[B39] Maher F. Vannucci S. J. Simpson I. A. (1994). Glucose transporter proteins in brain. FASEB J. 8, 1003–1011. 10.1096/fasebj.8.13.7926364 7926364

[B40] Mink J. W. Blumenschine R. J. Adams D. B. (1981). Ratio of central nervous system to body metabolism in vertebrates: its constancy and functional basis. Am. J. Physiol. Regul. Integr. Comp. Physiol. 241, R203–R212. 10.1152/ajpregu.1981.241.3.r203 7282965

[B41] Mishra D. Miyamoto T. Rezenom Y. H. Broussard A. Yavuz A. Slone J.. (2013). The molecular basis of sugar sensing in drosophila larvae. Curr. Biol. 23, 1466–1471. 10.1016/j.cub.2013.06.028 23850280 PMC4294765

[B42] Nishizaki T. Kammesheidt A. Sumikawa K. Asada T. Okada Y. (1995). A sodium- and energy-dependent glucose transporter with similarities to SGLT1-2 is expressed in bovine cortical vessels. Neurosci. Res. 22, 13–22. 10.1016/0168-0102(95)00876-U 7792078

[B43] Nishizaki T. Matsuoka T. (1998). Low glucose enhances Na+/glucose transport in bovine brian artery endothelial cells. Stroke 29, 844–849. 10.1161/01.STR.29.4.844 9550521

[B44] Pasco M. Y. Léopold P. (2012). High sugar-induced insulin resistance in Drosophila relies on the lipocalin Neural Lazarillo. PLoS ONE 7:e36583. 10.1371/journal.pone.0036583 22567167 PMC3342234

[B45] Patching S. G. (2016). Glucose transporters at the blood-brain barrier: function, regulation and gateways for drug delivery. Mol. Neurobiol. 54, 1046–1077. 10.1007/s12035-015-9672-6 26801191

[B46] Rehni A. K. Dave K. R. (2018). Impact of hypoglycemia on brain metabolism during diabetes. Mol. Neurobiol. 55, 9075–99088. 10.1007/s12035-018-1044-6 29637442 PMC6179939

[B47] Rodrigues F. Thuma L. Klämbt C. (2012). The regulation of glial-specific splicing of Neurexin IV requires HOW and Cdk12 activity. Development 139, 1765–1776. 10.1242/dev.074070 22461565

[B48] Rossi A. Kontarakis Z. Gerri C. Nolte H. Hölper S. Krüger M.. (2015). Genetic compensation induced by deleterious mutations but not gene knockdowns. Nature 524, 230–233. 10.1038/nature14580 26168398

[B49] Schmidt I. Thomas S. Kain P. Risse B. Naffin E. Klämbt C. (2012). Kinesin heavy chain function in Drosophila glial cells controls neuronal activity. J. Neurosci. 32, 7466–7476. 10.1523/JNEUROSCI.0349-12.2012 22649226 PMC6703594

[B50] Simpson I. A. Appel N. M. Hokari M. Oki J. Holman G. D. Maher F.. (1999). Blood-brain barrier glucose transporter: effects of hypo- and hyperglycemia revisited. J. Neurochem. 72, 238–247. 10.1046/j.1471-4159.1999.0720238.x 9886075

[B51] Simpson I. A. Vannucci S. J. DeJoseph M. R. Hawkins R. A. (2001). Glucose transporter asymmetries in the bovine blood-brain barrier. J. Biol. Chem. 276, 12725–12729. 10.1074/jbc.M010897200 11278779

[B52] Sivitz W. DeSautel S. Walker P. S. Pessin J. E. (1989). Regulation of the glucose transporter in developing rat brain. Endocrinology 124, 1875–1880. 10.1210/endo-124-4-1875 2924729

[B53] Stephan R. Grevelhörster A. Wenderdel S. Klämbt C. Bogdan S. (2008). Abi induces ectopic sensory organ formation by stimulating EGFR signaling. Mech. Dev. 125, 183–195 10.1016/j.mod.2007.12.002 18221859

[B54] Stork T. Engelen D. Krudewig A. Silies M. Bainton R. J. Klambt C. (2008). Organization and function of the blood brain barrier in Drosophila. J. Neurosci. 28, 587–597. 10.1523/JNEUROSCI.4367-07.2008 18199760 PMC6670337

[B55] Sulem P. Helgason H. Oddson A. Stefansson H. Gudjonsson S. A. Zink F.. (2015). Identification of a large set of rare complete human knockouts. Nat. Genet. 47,448–452. 10.1038/ng.3243 25807282

[B56] Takanaga H. Chaudhuri B. Frommer W. B. (2008). GLUT1 and GLUT9 as major contributors to glucose influx in HepG2 cells identified by a high sensitivity intramolecular FRET glucose sensor. Biochim. Biophys. Acta Biomembr. 1778, 1091–1099. 10.1016/j.bbamem.2007.11.015 18177733 PMC2315637

[B57] Vemula S. Roder K. E. Yang T. Bhat G. J. Thekkumkara T. J. Abbruscato T. J. (2009). A functional role for sodium-dependent glucose transport across the blood-brain barrier during oxygen glucose deprivation. J. Pharmacol. Exp. Ther. 328, 487–495. 10.1124/jpet.108.146589 18981287 PMC2630371

[B58] Volkenhoff A. Hirrlinger J. Kappel J. M. Klämbt C. Schirmeier S. (2018). Live imaging using a FRET glucose sensor reveals glucose delivery to all cell types in the Drosophila brain. J. Insect Physiol. 106, 55–64. 10.1016/j.jinsphys.2017.07.010 28733241

[B59] Volkenhoff A. Weiler A. Letzel M. Stehling M. Klämbt C. Schirmeier S. (2015). Glial glycolysis is essential for neuronal survival in Drosophila. Cell Metab. 22, 437–447. 10.1016/j.cmet.2015.07.006 26235423

[B60] Weiler A. Volkenhoff A. Hertenstein H. Schirmeier S. (2017). Metabolite transport across the mammalian and insect brain diffusion barriers. Neurobiol. Dis. 107, 15–31. 10.1016/j.nbd.2017.02.008 28237316

[B61] Weiszmann R. Hammonds A. S. Celniker S. E. (2009). Determination of gene expression patterns using high-throughput RNA *in situ* hybridization to whole-mount Drosophila embryos. Nat. Protoc. 4, 605–618. 10.1038/nprot.2009.55 19360017 PMC2780369

[B62] Wyatt G. R. Kalf G. F. (1957). The chemistry of insect hemolymph: II. Trehalose and other carbohydrates. J. Gen. Physiol. 40, 833–847. 13439163 10.1085/jgp.40.6.833 PMC2147581

[B63] Yildirim K. Petri J. Kottmeier R. Klämbt C. (2019). Drosophila glia: few cell types and many conserved functions. Glia 67, 5–26. 10.1002/glia.23459 30443934

[B64] Yu A. S. Hirayama B. A. Timbol G. Liu J. Diez-Sampedro A. Kepe V.. (2013). Regional distribution of SGLT activity in rat brain *in vivo. Am. J. Physiol*. Cell Physiol. 304, C240–C247. 10.1152/ajpcell.00317.2012 23151803 PMC3566441

