# Corrigendum: Plasticity of carbohydrate transport at the blood-brain barrier

**DOI:** 10.3389/fnbeh.2024.1443912

**Published:** 2024-07-19

**Authors:** Ellen McMullen, Astrid Weiler, Holger M. Becker, Stefanie Schirmeier

**Affiliations:** ^1^Department of Biology, Institute of Zoology, Technische Universität Dresden, Dresden, Germany; ^2^Division of General Zoology, Department of Biology, University of Kaiserslautern, Kaiserslautern, Germany

**Keywords:** blood-brain barrier, carbohydrate transport, compensatory mechanisms, transporter regulation, transport dynamics

In the published article, there was an error in the Funding statement. A funding source was missing in the original text: “The work was supported by grants of the DFG to SS (SFB1009, SCHI 1380/2-1)”. The correct Funding statement appears below.

