# Supplementary figures and images for: Plasticity of Carbohydrate Transport at the Blood-Brain Barrier
Source: Front Behav Neurosci. 2021 Jan 22;14:612430. doi: 10.3389/fnbeh.2020.612430 (PMC7863721; doi:10.3389/fnbeh.2020.612430)

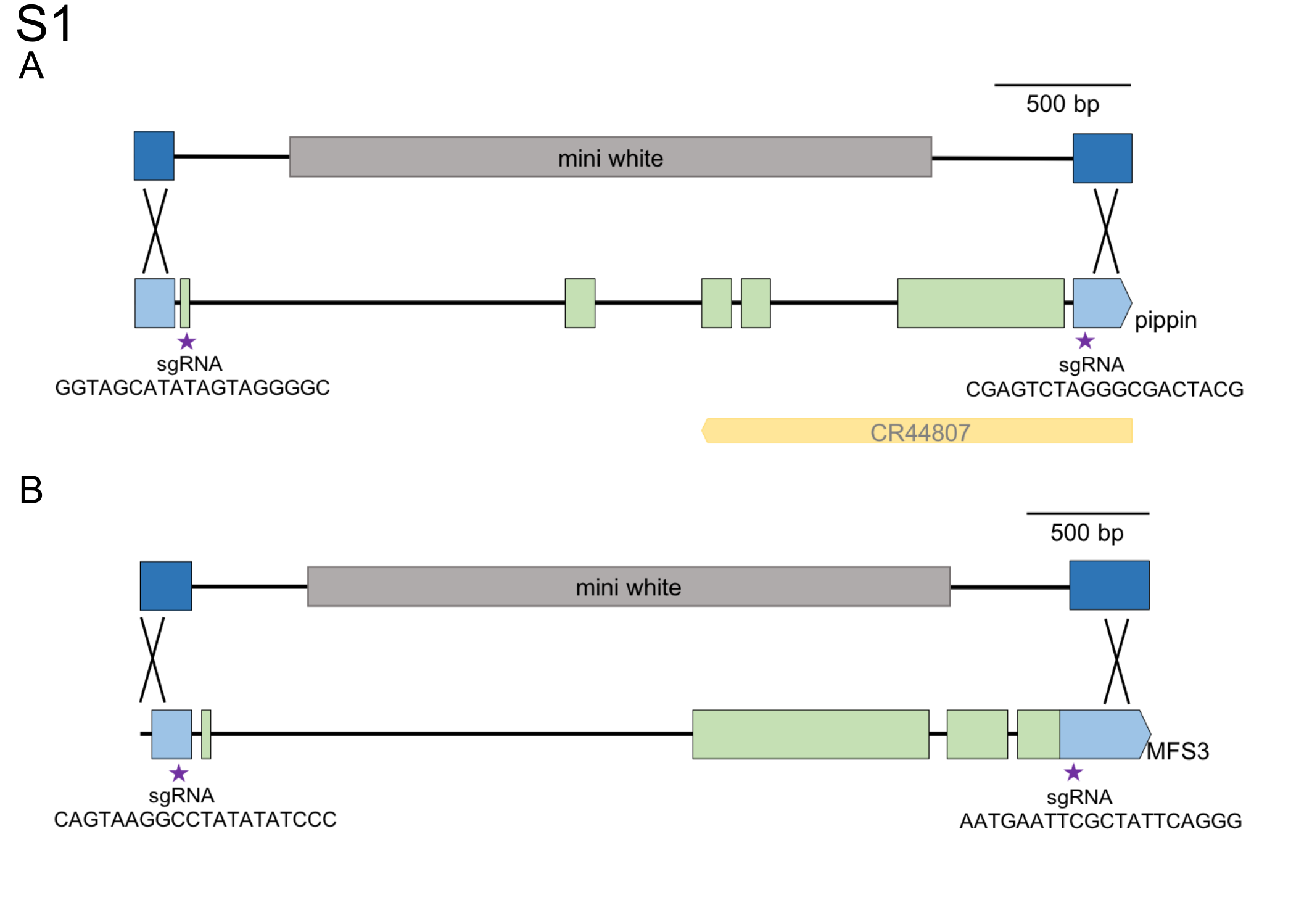

Supplement: Supplementary file 1 [file Image_1.TIF]

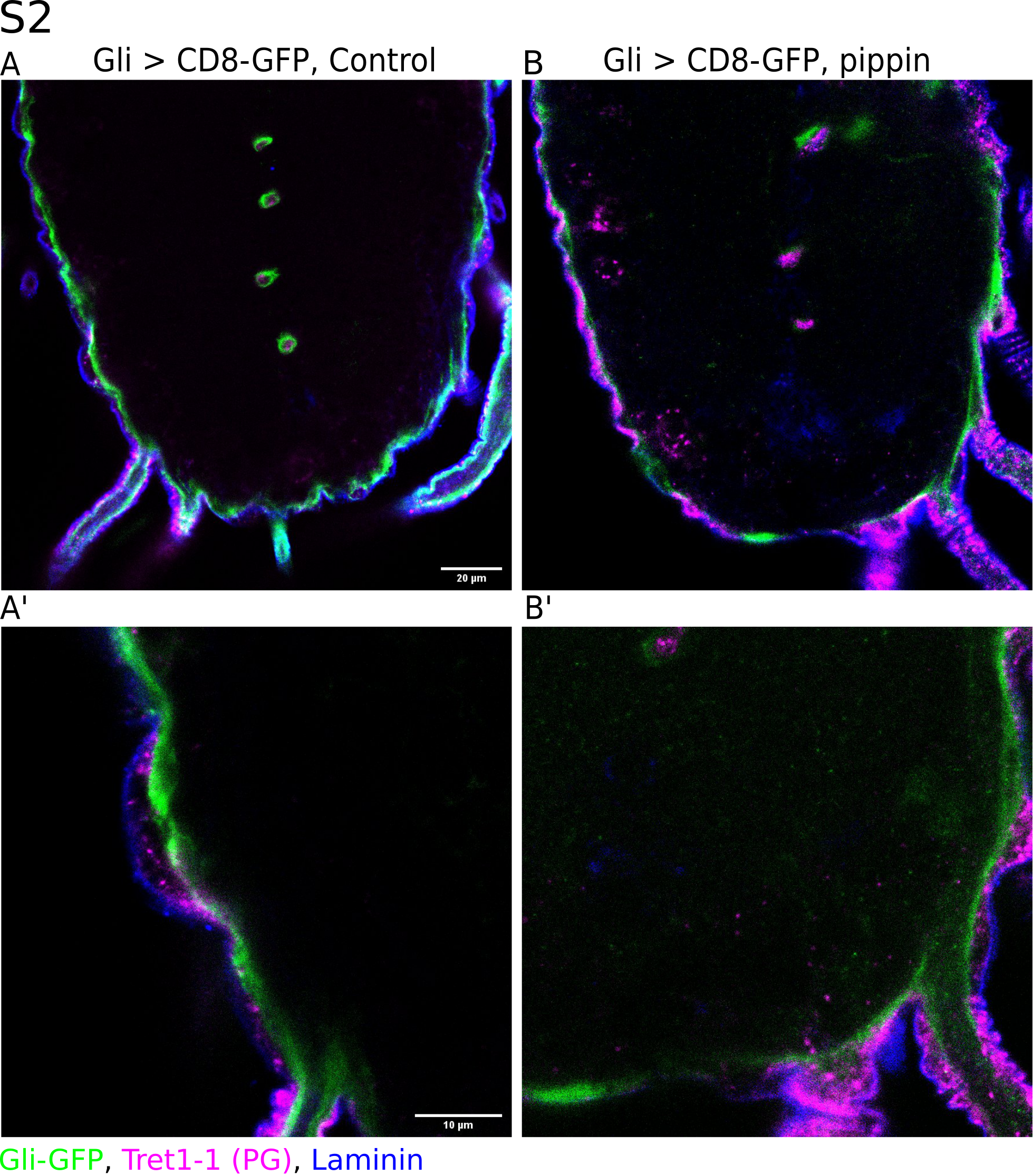

Supplement: Supplementary file 2 [file Image_2.TIF]
