# Supplementary material for: Plasticity of Carbohydrate Transport at the Blood-Brain Barrier
Source: Front Behav Neurosci. 2021 Jan 22;14:612430. doi: 10.3389/fnbeh.2020.612430 (PMC7863721; doi:10.3389/fnbeh.2020.612430)
Supplement: Supplementary file 3 [file Data_Sheet_1.docx]

**Supplementary information**

**Supplementary Figure 1: Generation of CRISPR-induced null mutants**

**(A)** Scheme illustrating CRISPR-mediated homologous recombination-induced exchange of the coding sequence of *pippin* against a mini-white. The sgRNA target sites are indicated. Dark blue: homology arm, light blue: UTR, green: coding exon, CR44807: encodes a non-coding RNA. **(B)** Scheme illustrating CRISPR-mediated homologous recombination-induced exchange of the coding sequence of *Mfs3* against a mini-white. The sgRNA target sites are indicated. Dark blue: homology arm, light blue: UTR, green: coding exon.

**Supplementary Figure 2: Localisation of Tret1-1 in pippin null mutants**

**(A, A’)** Tret1-1 is localized in the perineural glial cells of control animals. **(B, B’)** Upon loss of Pippin, Tret1-1 is more abundant in the perineural glia but does not mislocalize to the subperineurial glia.

**Supplementary Table 1: RNA interference screen**

Results of the RNAi screen that was performed to identify putative carbohydrate transporters essential in the glial cells. The RNAi lines used are indicated. The phenotype of panglial knockdown at 25°C is indicated in terms of viability and adult behavioral phenotype. jumper: the animals cannot fly; walker: the animals can neither fly nor jump properly; sitter: the animals hardly move at all; wt: wild typic.

| **CG #** | **Gene name** | **dsRNA construct** | **viability** | **locomotor capacity** |
| --- | --- | --- | --- | --- |
| CG10069 |  | 108635 | viable | wt |
| CG10486 |  | 107903 | viable | wt |
| CG1086 | glucose transporter 1 | 13328 | viable | walker |
|  |  | 101365 | viable | wt |
| CG10960 | nebulosa | 34598 | viable | wt |
| CG11537 |  | 4118 | viable | wt |
| CG11880 | Choline transporter-like 2 | 22869 | viable | wt |
|  |  | 106968 | viable | wt |
| CG1213 |  | 6487 | viable | Wt |
| CG12783 |  | 101110 | viable | wt |
| CG1311 | Choline transporter-like 1 | 13480 | viable | wt |
| CG13610 | organic cation transporter 2 | 1177 | viable | walker |
|  |  | 48870 | viable | walker |
|  |  | 106681 | viable | wt |
| CG1380 | sugar transporter 4 | 44934 | viable | wt |
| CG14160 |  | 11157 | viable | wt |
|  |  | 104744 | viable | wt |
| CG14605 |  | 105310 | viable | wt |
| CG14606 |  | 100903 | viable | wt |
| CG14855 |  | 40906 | viable | wt |
| CG14856 |  | 101004 | viable | wt |
| CG15096 |  | 39462 | viable | wt |
|  |  | 103956 | viable | wt |
| CG15406 |  | 105077 | viable | wt |
| CG15408 |  | 43816 | viable | wt |
|  |  | 43817 | viable | wt |
| CG15553 |  | 6866 | viable | wt |
|  |  | 104514 | viable | wt |
| CG16727 |  | 33363 | viable | wt |
|  |  | 100852 | viable | wt |
| CG17036 |  | 2869 | viable | wt |
|  |  | 100656 | viable | wt |
| CG17751 |  | 8619 | viable | wt |
|  |  | 50469 | viable | wt |
| CG17752 |  | 26749 | viable | wt |
|  |  | 50274 | viable | wt |
|  |  | 106787 | viable | wt |
| CG17929 |  | 100093 | viable | wt |
| CG17930 |  | 108912 | viable | wt |
| CG17975 | sugar transporter 2 | 102028 | viable | wt |
| CG17976 | sugar transporter 3 | 4009 | viable | wt |
| CG2675 | UDP-galactose transporter | 100803 | viable | wt |
| CG30035 | trehalose transporter 1-1 | 52360 | viable | sitter |
|  |  | 103045 | viable | wt |
| CG30345 |  | 103652 | viable | wt |
| CG3057 | congested-like trachea | 106089 | viable | wt |
| CG31100 |  | 42627 | viable | jumper |
| CG3168 |  | 48010 | viable | wt |
| CG32053 |  | 107225 | viable | wt |
| CG32054 |  | 107214 | viable | wt |
| CG3285 |  | 52669 | viable | wt |
|  |  | 105747 | viable | wt |
| CG33181 |  | 103142 | viable | wt |
| CG33233 |  | 106897 | viable | wt |
| CG33281 |  | 7273 | viable | wt |
| CG33282 |  | 100325 | viable | wt |
| CG33933 | I'm not dead yet 2 | 51048 | viable | wt |
| CG33934 |  | 50699 | viable | wt |
| CG3774 | ER GDP-fucose transporter | 30238 | viable | wt |
| CG3790 | Beta-alanine transporter | 4667 | viable | wt |
|  |  | 108223 | viable | wt |
| CG3853 | glucose transporter type 3 | 100253 | viable | wt |
|  |  | 3853R-1 | viable | wt |
| CG3874 | fringe connection | 21469 | viable | wt |
|  |  | 47542 | viable | wt |
|  |  | 107816 | viable | wt |
| CG42269 |  | 100344 | viable | wt |
| CG42825 |  | 9017 | viable | wt |
| CG4324 |  | 37211 | viable | wt |
|  |  | 109433 | viable | wt |
| CG4484 | Solute carrier family 45 member 1 | 5172 | viable | wt |
| CG4607 |  | 5450 | lethal | - |
| **CG4726** | **major facilitator superfamily transporter 3** | **4726R-3** | **lethal** | **-** |
| **CG4797** | **pippin** | **10598** | **lethal (few escapers)** | **-** |
| CG4994 | Mitochondrial phosphate carrier protein 2 | 101316 | late pupal lethal | - |
| CG5078 |  | 8085 | late larval/pupal lethal | - |
| CG5592 |  | 110489 | viable | wt |
| CG5760 | tetracycline resistance | 110473 | viable | wt |
| CG5802 | medial glomeruli | 6800 | viable | wt |
| CG6006 |  | 11639 | viable | wt |
|  |  | 106513 | viable | wt |
| CG6126 |  | 7326 | viable | wt |
| CG6231 |  | 42655 | viable | walker/jumper |
|  |  | 105194 | viable | wt |
| CG6331 | Organic cation transporter | 6782 | viable | wt |
|  |  | 47133 | viable | wt |
| CG6356 |  | 101866 | late pupal lethal | - |
|  |  | 28745 | late pupal lethal | - |
|  |  | 23013 | late pupal lethal | - |
| CG6484 |  | 109481 | viable | wt |
| CG6574 |  | 40903 | viable | - |
|  |  | 110570 | viable | wt |
| CG6782 | scheggia | 50713 | viable | wt |
| CG6901 |  | 104673 | viable | wt |
| CG7009 | tRNA methyltransferase 7-34 | 23013 | viable | wt |
| CG7084 |  | 35677 | larval lethal | - |
|  |  | 12162 | larval lethal | - |
| CG7333 |  | 8617 | viable | wt |
|  |  | 101790 | viable | wt |
| CG7342 |  | 48001 | viable | wt |
|  |  | 7342R-1 | viable | wt |
| CG7458 |  | 108770 | viable | wt |
| CG7623 | slalom | 12148 | viable | wt |
| CG7882 |  | 109918 | viable | wt |
| CG8234 | Trehalose transporter 1-2 | 40980 | viable | wt |
|  |  | 49889 | viable | wt |
| CG8249 |  | 50438 | viable | wt |
|  |  | 106077 | viable | wt |
| CG8654 |  | 4715 | viable | wt |
|  |  | 100112 | viable | wt |
| CG8714 | sugar transporter 1 | 104983 | viable | wt |
| CG8717 | saliva | 104666 | viable | wt |
| CG8837 |  | 100670 | viable | wt |
| CG8925 |  | 37461 | viable | wt |
| CG9090 | Mitochondrial phosphate carrier protein 1 | 101848 | viable | wt |
| CG9317 | Carcinine transporter | 9321 | viable | wt |
| CG9620 | neuronally altered carbohydrate | 105410 | viable | wt |
| CG9657 |  | 43922 | semi lethal/ adult lethal | - |
|  |  | 107361 | viable | wt |
| CG1732 | GABA transporter | 106638 | lethal | - |
|  |  | 29422 | lethal | - |
| CG5485 | Prestin | 5341 | viable | wt |
|  |  | 50706 | viable | wt |
| CG9903 |  | 42690 | viable | wt |
| CG16700 |  | 110058 | viable | wt |
| CG11655 |  | 9131 | viable | wt |
